# Arc of Buhler Pseudoaneurysm Causing Fatal Retroperitoneal Hemorrhage; A Rare Case Report and Discussion of Relevant Literature

**DOI:** 10.29252/beat-070215

**Published:** 2019-04

**Authors:** Saptarshi Biswas, Shekhar Gogna

**Affiliations:** 1 *Department of Trauma and Acute Care Surgery, Forbes Hospital, Allegheny Health Network, Pennsylvania. USA*; 2 *Department of General Surgery, Westchester University Medical Center, Valhalla, New York, USA *

**Keywords:** Pseudoaneurysm, Fatal hemorrhage, Retroperitoneum, Arc of Buhler (AOB)

## Abstract

Identification of any variant anatomy prior to surgery is as essential as having knowledge of normal anatomy. These surprises bring on many challenges along with as they can be fatal. We encountered a case of patient who succumbed down to an unrecognized rare mesenteric vasculature variant known as “Arc of Buhler” (AOB) which is a persistent embryonic ventral anastomosis between the Celiac trunk and the Superior mesenteric artery. It is usually asymptomatic and found incidentally after evaluation for other pathologies. We herein report a pseudoaneurysm of Arc of Buhler being surgically managed after massive retroperitoneal hemorrhage. Unfortunately, the patient did not survive the procedure and passed away. AOB aneurysms present formidable risks to patients and diagnostic and therapeutic challenges to physicians. They are rare and require high index of suspicion on radiographic imaging. Present case reports underscore the importance of identifying it and treating it regardless of the size.

## Introduction

The gastrointestinal system is mainly supplied by celiac trunk, superior mesenteric artery (SMA), and inferior mesenteric Artery (IMA) [1]. There are numerous anatomical variants and anastomoses have been reported between these arteries. There is important clinical significance of these collaterals as they protect the bowel from potential infarction and ischemia [2]. The Arc of Buhler (AOB), which is one of these variants and anastomoses, is a persistent embryonic ventral anastomosis between the Celiac trunk and the Superior mesenteric artery [3]. This anatomical variant has an incidence rate of 1–4% [4]. Most commonly identified on imaging performed on patients for some other reasons. Aneurysms of AOB are mentioned in literature especially associated with Celiac artery stenosis due to median arcuate ligament syndrome or atheroma [5]. There is a case report with AOB as causing obstructive jaundice [6]. Our patient presented with massive retroperitoneal bleed after rupture of AOB. We reviewed the available literature on AOB, its implication and management modalities in this case report. 

## Case Presentation

**Fig. 1:**
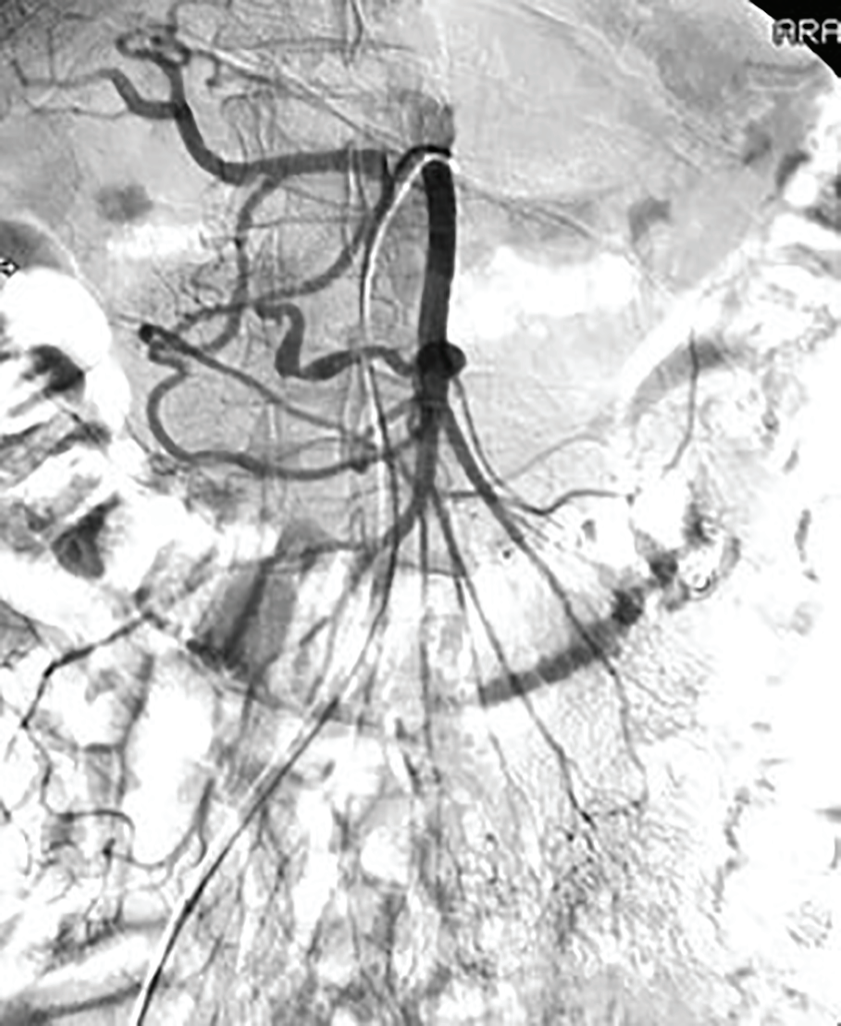
Digital subtraction angiography (DSA) of the mesenteric artery demonstrating pseudoaneurysm of the Arc of Buhler (AOB) (Arrow)

**Fig. 2:**
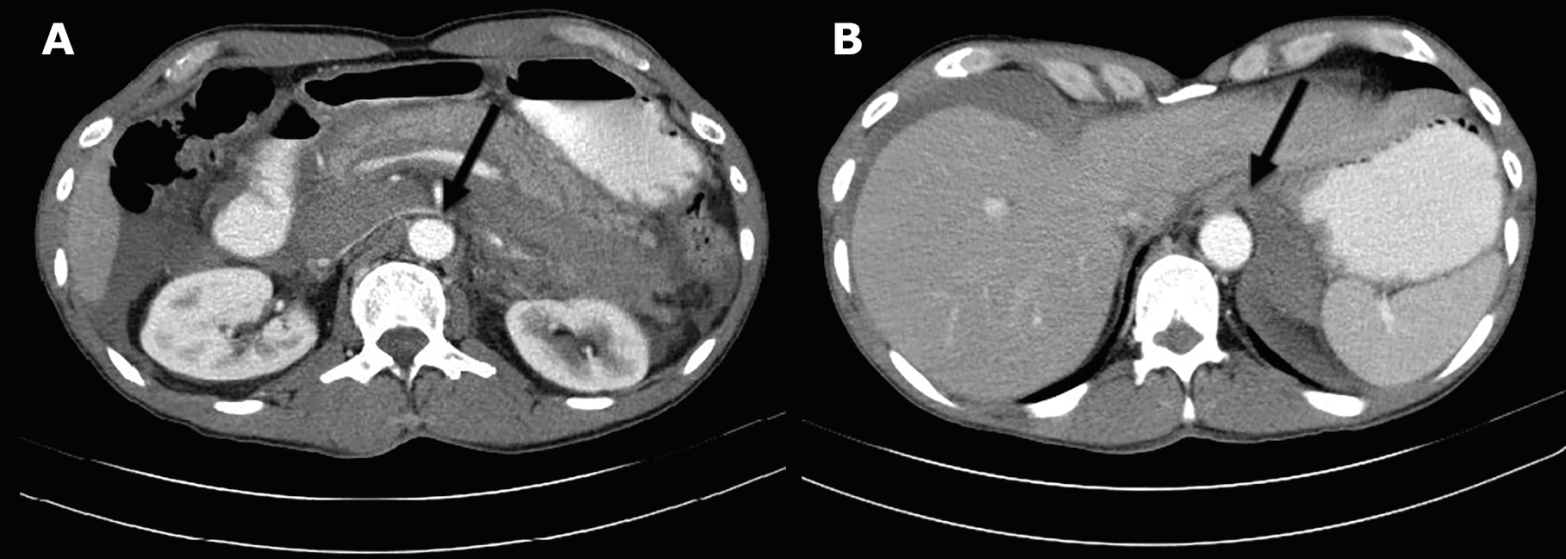
Axial computed tomography (CT) of the abdomen demonstrating retroperitoneal bleeding in the patient (A; Arrow); axial CT-scan of the abdomen demonstrating free air around the liver (B; Arrow)

A 55-year-old man presented to the emergency department (ED) with acute onset abdominal pain started insidiously approximately 1-hour prior to presentation. He was discharged only a week before from the same hospital when he was admitted with acute abdominal pain when he underwent a CT-angiogram and a digital subtraction angiography (DSA) of the abdomen, which showed incidental finding of Arc of Buhler (Figure 1). There was discussion regarding management of this incidental finding and decision was taken not to embolize it by intervention radiology as it was incidental finding and pain was not attributed to that.  He was subsequently discharged after observation in the hospital for a couple of days when his labs remained steady. During this admission the patient was alert and oriented in time and place. Abdomen was soft to palpation and tender on deep palpation without peritoneal signs on clinical exam. The abdominal radiography showed partial small bowel obstruction. The CT-scan of the abdomen showed big retroperitoneal hematoma and free fluid around the liver (Figure 2). Review of systems was grossly nonspecific on arrival to the ED except sharp abdominal pain. His past medical history was essentially negative for any chronic diseases; his surgical history consisted of inguinal hernia repair. After 40-min the patient had sudden cardiac arrest and cardiopulmonary resuscitation as per ACLS protocol was initiated. The patient was emergently intubated. A right subclavian central line was placed. Volume resuscitation was initiated, patient was started on pharmacologic presser agents and massive transfusion protocol was set up. We were working on provisional diagnosis of ruptured Arc of Buhler. Seven units of packed red blood cells (PRBC), 6 units of fresh frozen plasma (FFP) and 2 units of platelets were transfused. His abdomen was massively distended. His vitals were recorded as BP 90/52; HR 120; oxygen saturation at 90%. His labs showed Hb/Hct of 4.8/14.8; and platelet of 90. His serum chemistry was normal at this time with Na 135; K 3.6; Cl 93; CO2 21; BUN 17; Cr 0.1. The patient was taken to the operating room (OR) emergently. A midline trauma incision was made extending from the xiphisternum to the symphysis pubis. Two cell saving suctions were used. About 2L of hemoperitoneum and clotted blood were suctioned out. Massive transfusion was continued intraoperatively. Four quadrant packing was used to temporize the bleeding. A window through the gastrohepatic ligament was used to clamp the supra celiac aorta. There was large bulging retroperitoneal hematoma with gross distortion of anatomy. The spleen was ruptured. There was significant generalized diffuse oozing suggestive of disseminated intravascular coagulation (DIC). We used packs and hemostatic gauze were placed to tamponade the bleeding. The abdomen was left open with an Abthera and Vaccum assisted closure (VAC) with a plan to return to the OR when the acidosis, hypothermia and coagulopathy is corrected. The patient was transferred to the PACU in critical condition. Early next day morning the patient had a sudden cardiorespiratory arrest. ACLS protocol along with CPR was initiated. However, the attempt was futile and the patient was pronounced dead after all resuscitative efforts.

## Discussion

There are several procedures, both surgical and interventional, that require detailed knowledge of the hepatic and celiac trunk variations for example hepatic and pancreatic surgery, liver transplantation and intra-arterial infusion of chemotherapy procedures. 

The AOB was first described by Buhler and Tandler in 1904 [7]. Tandler’s linear regression concept during embryogenesis explains the formation of AOB. The SMA and the celiac trunk arise during the prenatal period from the 10th and 13th segmental arteries of aorta, respectively. These segmental arteries are connected by a ventral anastomosis, which usually regresses. AOB arises from failure of this regression of this persistent ventral communication between the SMA and the celiac trunk or one of its branches [8]. There are three main primary collateral pathways between Celiac artery and SMA. These are gastro duodenal artery, pancreotico-duodenal arteries, and dorsal pancreatic artery [2]. AOB is the least common collateral amongst these pathways. 

This condition is usually detected incidentally in asymptomatic patients undergoing imaging for other reasons as well as in symptomatic patients with celiac trunk stenosis related to arterial sclerosis or median arcuate ligament syndrome [9]. This vascular anomaly is very rarely encountered, and the rupture of these AOB aneurysms may lead to a life-threatening condition. Our patient presented with fatal massive retroperitoneal bleed due to ruptured aneurysm of AOB. Although the management of this aneurysm is unclear. With advances in interventional radiology, coil embolization is the first choice of treatment [5]. Importantly, embolization of this aneurysm may result in a significantly greater blood flow at other vessels such as a pancreaticoduodenal artery resulting in recurrence of the aneurysm. Hence, careful and long-term follow-up is necessary [10]. Table 1 depicts some of the case reports highlighting the presentation and management deployed for this rare pathology. There are two cases of surgical resection of aneurysm, Myers described the surgical resection of an AOB aneurysm with SMA to splenic artery bypass using saphenous vein in a patient with neurofibromatosis type 1 [11]. 

Kugai described aneurysmectomy of 3-cm saccular AOB in a patient with a celiac artery occlusion. Surgery was performed without revascularization of the Celiac artery after attempts at failed embolization [12]. Closely related differential diagnosis to the AOB is the pancreaticoduodenal artery arcade (PDAA). The PDAA, consisting of both the superior (SPDA, anterior and posterior) and inferior (IPDA, anterior and posterior) pancreaticoduodenal arteries. This pathway provides the normal collateral flow pathway between the SMA and celiac arterial system [5]. The importance of early identification of AOB lies in the fact that it might show up as a surprise while doing surgery on pancreas, liver or stomach [13]. In our case patient died because of rupture of AOB pseudo aneurysm. 

In conclusion, AOB aneurysms present formidable risks to patients and diagnostic and therapeutic challenges to physicians. They are rare and require high index of suspicion on radiographic imaging. Present case reports underscore the importance of identifying it and treating it regardless of the size. 

**Table 1:** Various case reports highlighting presentation and management of patents with Arc of Buhler (AOB)

**Author/Year**	**Patient age/sex**	**Presentation**	**Treatment**
**Myers JL /1998 [** 11 **]**	39 year/Female	Urinary frequency	Elective open surgical excision
**Dubel GJ/2007 [** 7 **]**	54 year/Male	Aneurysm presented with rising Liver function tests	Transcatheter embolization in Interventional radiology
**Sugihara F /2016 [** 10 **]**	35 year/Male	Median arcuate ligament syndrome after Celiac artery stenosis	Transcatheter arterial embolization
**Ochoa/2016 [** 4 **]**	58 Year/Male	Aneurysm presented with Falling Hematocrit after Pancreateticoduodenectomy	Transcatheter embolization in Interventional radiology
**Kageyama Y/2016 [** 13 **]**	74 year/Female	Discovered during Pancreateticoduodenectomy	Observation
**McCracken et al., 2018 [** 14 **]**	69 year/Male	Discovered after Pancreateticoduodenectomy	Observation
**Present case report**	55 year/Male	Readmitted after hemorrhagic shock due to rupture of Aneurysm	Death

## Conflict of Interest:

None declared. 
